# Molecular mechanisms regulating natural menopause in the female ovary: a study based on transcriptomic data

**DOI:** 10.3389/fendo.2023.1004245

**Published:** 2023-07-24

**Authors:** Quan Liu, Fangqin Wei, Jiannan Wang, Haiyan Liu, Hua Zhang, Min Liu, Kaili Liu, Zheng Ye

**Affiliations:** ^1^ Binhu Hospital, Hefei First People’s Hospital, Hefei, Anhui, China; ^2^ State Key Laboratory of Bioelectronics, Southeast University, Nanjing, China

**Keywords:** menopause, scRNA-seq, bulk-RNA-seq, natural ageing, Granulosa

## Abstract

**Introduction:**

Natural menopause is an inevitable biological process with significant implications for women's health. However, the molecular mechanisms underlying menopause are not well understood. This study aimed to investigate the molecular and cellular changes occurring in the ovary before and after perimenopause.

**Methods:**

Single-cell sequencing data from the GTEx V8 cohort (30-39: 14 individuals; 40-49: 37 individuals; 50-59: 61 individuals) and transcriptome sequencing data from ovarian tissue were analyzed. Seurat was used for single-cell sequencing data analysis, while harmony was employed for data integration. Cell differentiation trajectories were inferred using CytoTrace. CIBERSORTX assessed cell infiltration scores in ovarian tissue. WGCNA evaluated co-expression network characteristics in pre- and post-perimenopausal ovarian tissue. Functional enrichment analysis of co-expression modules was conducted using ClusterprofileR and Metascape. DESeq2 performed differential expression analysis. Master regulator analysis and signaling pathway activity analysis were carried out using MsViper and Progeny, respectively. Machine learning models were constructed using Orange3.

**Results:**

We identified the differentiation trajectory of follicular cells in the ovary as ARID5B+ Granulosa -> JUN+ Granulosa -> KRT18+ Granulosa -> MT-CO2+ Granulosa -> GSTA1+ Granulosa -> HMGB1+ Granulosa. Genes driving Granulosa differentiation, including RBP1, TMSB10, SERPINE2, and TMSB4X, were enriched in ATP-dependent activity regulation pathways. Genes involved in maintaining the Granulosa state, such as DCN, ARID5B, EIF1, and HSP90AB1, were enriched in the response to unfolded protein and chaperone-mediated protein complex assembly pathways. Increased contents of terminally differentiated HMGB1+ Granulosa and GSTA1+ Granulosa were observed in the ovaries of individuals aged 50-69. Signaling pathway activity analysis indicated a gradual decrease in TGFb and MAPK pathway activity with menopause progression, while p53 pathway activity increased. Master regulator analysis revealed significant activation of transcription factors FOXR1, OTX2, MYBL2, HNF1A, and FOXN4 in the 30-39 age group, and GLI1, SMAD1, SMAD7, APP, and EGR1 in the 40-49 age group. Additionally, a diagnostic model based on 16 transcription factors (Logistic Regression L2) achieved reliable performance in determining ovarian status before and after perimenopause.

**Conclusion:**

This study provides insights into the molecular and cellular mechanisms underlying natural menopause in the ovary. The findings contribute to our understanding of perimenopausal changes and offer a foundation for health management strategies for women during this transition.

## Introduction

Population aging is a definite trend of global population change (1). For women, there are also more health risks associated with aging because they live seven years longer than men on average (2). Numerous studies have shown that one of the unique influences of ageing on women’s health risks is the female menopause (3, 4).

Menopause is generally referred to as perimenopausal syndrome, a group of syndromes in which women experience fluctuations or decreases in sex hormones around the time of menopause, mainly due to dysfunction of the autonomic nervous system, accompanied by neuropsychological symptoms (5, 6). Natural menopause is a process that occurs naturally in the vast majority of women as they age (7, 8). The main manifestation of natural menopause is the exhaustion of follicles in the ovaries or the loss of response to gonadotropins in the remaining follicles, which no longer develop and secrete estrogen and cannot stimulate the growth of the endometrium, leading to menopause (9, 10). Perimenopause occurs mainly around the age of 50, with a global range of between 40 and 60 years (11, 12). During perimenopause, women may experience a range of menopausal syndromes, such as hot flushes and night sweats, insomnia, vaginal dryness and mood disorders (13). Although these symptoms are not life-threatening, they can substantially affect the quality of life and the physical and mental health of perimenopausal women. Despite the profound impact of perimenopausal syndrome on women’s health, the main molecular and cytological mechanisms are not currently studied. Because menopause is a unique physiological phenomenon of human beings (14), model animals cannot provide good research materials, so the research on the molecular mechanism of female menopause is still in the enlightenment stage.

In this study, we used the transcriptome database of female ovarian tissues from the GTEx V8 database (15), combined with single-cell sequencing data of ovarian tissues, to delve into the transcriptome characteristics of female ovaries before and after perimenopause (30–39, 40–49, 50–59) and to reveal the molecular and cytological mechanisms of changes in female ovaries before and after perimenopause. The results of this study provide a theoretical basis for research related to female menopausal syndromes. This study calls for more research teams to focus on basic research related to women’s menopausal health.

## Methods

### Data sources

**Figure 1:**
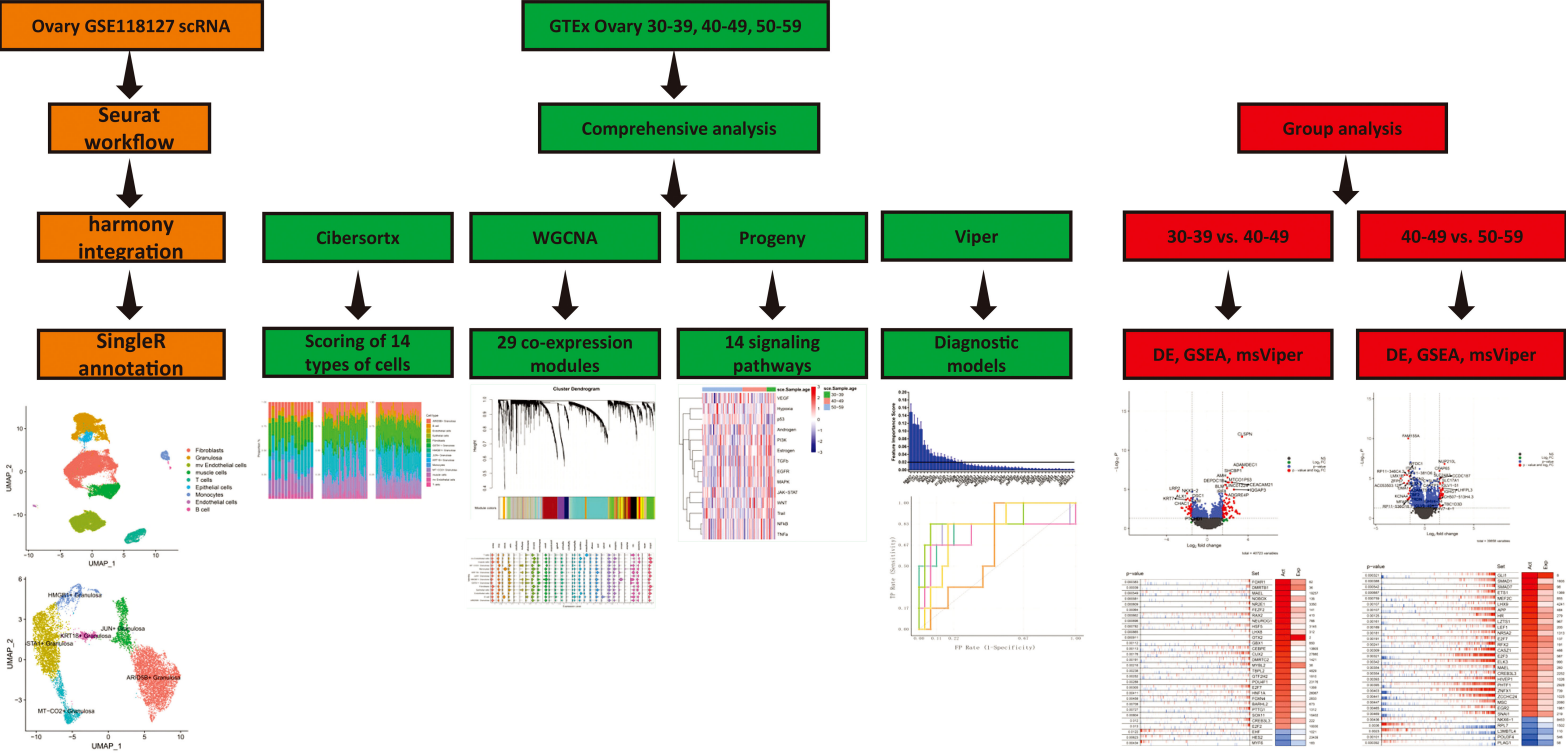
Work Flow.

All samples involved in this study were obtained from ovarian tissue samples in the GTEx V8 dataset. Clinical information for the samples is in Appendix. We screened out the age groups before and after perimenopause, and obtained 14 samples in the 30-39 age group, 37 samples in the 40-49 age group and 61 samples in the 50-59 age group. The single-cell sequencing data were derived from the GSE118127 (16) cohort, which included single-cell sequencing data from 36 normal ovarian tissues. The overall workflow is shown in **Figure 1**.

### Single-cell sequencing analysis

Seurat4.1 (17) was used for preliminary processing of single-cell sequencing data. The samples are preprocessed by SCTransform (18). Finally, a normalized expression profile matrix was obtained. Harmony was used to integrate 36 single-cell sequencing samples. RunPCA is used to perform linear dimensionality reduction of the data and find the main top 30 principal components. Use FindNeibors (ndims=1:30) to calculate the distance between samples, and use RunUMAP (ndims=1:30) to perform nonlinear dimensionality reduction on the samples. For differential expression analysis, we used the one vs. others strategy and used the FindAllMarker (Wilcoxon-Test) function to find the differential genes in different treatment groups. The differential genes obtained according to the screening conditions adj.P<0.01, abs(avg_log2FC)>0.5 were considered as differentially expressed genes. SingleR (19) was used to annotate cells. The CytoTRACE (20) algorithm was used with default parameters to compare the differentiation status of follicular cells in the dataset. The CytoTRACE algorithm enables robust reconstruction of cell differentiation trajectories. To explore the activation status of Estrogen-related signaling pathways in ovarian tissue cells, we collected 7 gene sets of Estrogen-related signaling pathways from the MSIGDB database (21)(GOBP_INTRACELLULAR_ESTROGEN_RECEPTOR_SIGNALING_PATHWAY,GOBP_NEGATIVE_REGULATION_OF_INTRACELLULAR_ESTROGEN_RECEPTOR_SIGNALING_PATHWAY,GOBP_REGULATION_OF_INTRACELLULAR_ESTROGEN_RECEPTOR_SIGNALING_PATHWAY,GOMF_ESTROGEN_16_ALPHA_HYDROXYLASE_ACTIVITY,GOMF_ESTROGEN_2_HYDROXYLASE_ACTIVITY,GOMF_NUCLEAR_ESTROGEN_RECEPTOR_BINDING,HP_ABNORMAL_CIRCULATING_ESTROGEN_LEVEL). Seurat function AddModuleScore was used to calculate signature scores for gene sets. VlnPlot was used to visualize the gene set scores.

### Impute cell fractions with CIBERSORTx

We prepared and uploaded the mixture datasets of 112 ovary transcriptome sequencing data according to the instructions with CIBERSORTx (22, 23). Then we chose the signature matrix we obtained from scRNA data. Since scRNA data was derived from 10xGenomics, we selected “S-mode” to batch correction. We set permutations to 500. Other parameters retained the default. After running CIBERSORTx, we obtained the relative proportions of 14 celltypes of ovary tissue in each sample with *p*-value measuring the confidence of the results for the deconvolution. Samples with *P* < 0.05 were included in a further study.

### Weighted gene co-expression network analysis

WGCNA (24) was used to analyze co-expressed module genes in ovarian samples. We calculated the Pearson correlation of each module with these scores based on age information included in the analysis. WGCNA algorithm allows the construction of scale-free networks between genes based on their expression information and then clustering of the closest genes. We selected genes with standard deviation >0.8 as input and merged genes with module distance <0.25. The enrichGO function in clusterprofileR (25) was used to perform functional enrichment analysis on genes with hub Gene (screening criteria Module Membership >0.7) in the gene set (**Appendix 1**).

### Differential expression analysis and functional enrichment analysis

DESeq2 (26) was used to analyze differentially expressed genes in the 20-29 and the 30-39 age group. ClusterprofileR was used to perform GSEA (27) (based on log2Foldchange ranking) on differentially expressed genes. Metascape (28) was used to analyze the function, Protein-Protein-Interaction network and coregulatory network of ageing-related genes.

### Master regulator analysis

Msviper (29) was used to analyze master regulators in groups 30-39, 40-49, 50-59. The regulatory network of transcription factors was first assessed by ARACNE-AP (30) based on 112 transcriptome samples (Appendix1). Then transcription factor activity was assessed for groups 40-49 and 30-39 using msviper. The same method was used to compare the 50-59 age group with the 40-49 age group. In addition, viper was used to assess transcription factor activity for each sample. This data is used for subsequent machine learning model building.

### Analysis of signaling pathway activity

PROGENy (31) is a resource that uses a large collection of signaling perturbation experiments that are available to the public to find a core of pathway-responsive genes that are the same for both humans and mice. With these and any statistical method, you can use bulk or single-cell transcriptomics to figure out how a pathway is working. We assessed the activity of 14 common signaling pathways in 126 skin samples using the R package decoupleR (32). First, we get the model constructed by the top500 genes through the get_progeny function. Then, the weights of the 14 signaling pathways in the 112 samples are evaluated using the average weight algorithm (run_wmean).

### Diagnostic models

Transcription factor activity matrices inferred using viper were used to construct a three-category diagnostic model. Orange3 (33) is used to build the machine learning model algorithm framework. Orange3 is an interactive machine learning platform. Through 1000 L1 regularized logistic regression, features with weights higher than 0.2 were screened for model construction. To find the most suitable machine learning model, we built SVM, Random Forest, Naive Bayes, Logistic Regression L2, Logistic Regression L1, Gradient Boosting 6 machine learning models are used for training and testing models. AUC, CA, F1, Precision, Recall are used to evaluate the performance of the model. ROC and Calibration curve were used to evaluate the diagnostic ability of the model.

### Statistics

R 4.10 was used to perform statistical analyses. the R package ggpubr (34) was used for statistical plots and statistical tests. enrichplot was used to plot the results of GSEA. P<0.05 was considered statistically significant. *P < 0.05; ** P < 0.01; *** P<0.005; **** P<0.001; ns Not Significant.

## Results

### Characteristics of ovarian tissue microenvironment at the single-cell level

**Figure 2:**
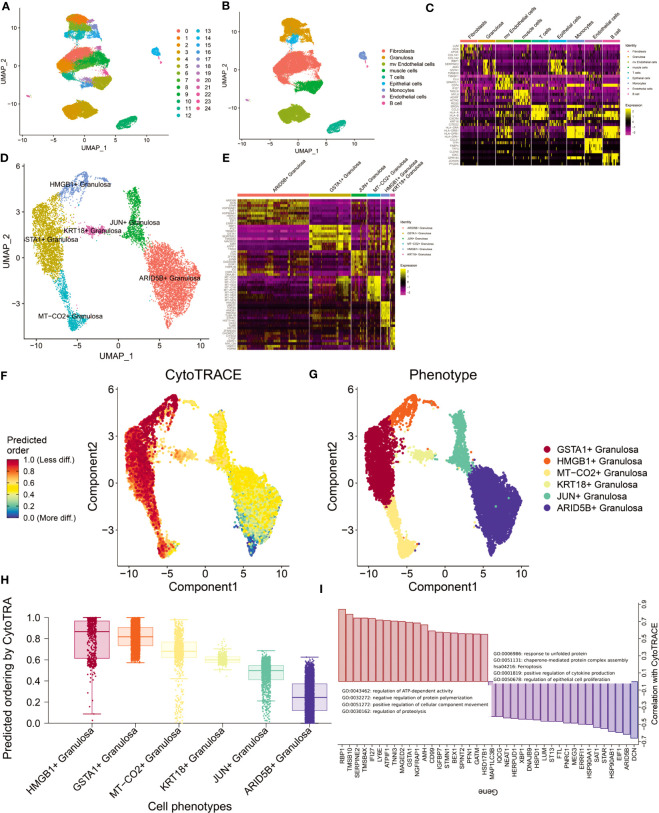
Analysis of single-cell sequencing data from ovarian tissue. **(A)** Louvain cluster yielded 25 clusters. **(B)** SingleR cell annotation (BlueprintEncoder as reference dataset) yielded 9 major cell types. **(C)** Differentially expressed genes (top5) of 9 cell types. **(D)** Granulosa performed in-depth single-cell clustering analysis and obtained a total of 6 clusters. **(E)** Differentially expressed genes (top10) of the six Granulosa subtypes. **(F)** Differentiation trajectories obtained by CytoTRACE. **(G)** Distribution of 6 Granulosa cell types. **(H)** Ranking of cell differentiation according to CytoTRACE. **(I)** Functional annotation of major regulatory genes and gene sets driving cell differentiation.

The GSE118127 cohort contains single-cell sequencing data from 36 normal ovarian tissues. Through the Seurat standard process (SCTransform+harmony), 24 clusters were obtained (louvain unsupervised clustering algorithm; **Figure 2A**). Cells were annotated by SingleR and 9 cell types were obtained (Fibroblasts, Granulosa, mv Endothelial cells, muscle cells, T cells, Epithelial cells, Monocytes, Endothelial cells, B cells; **Figure 2B**). By FindAllmarker(q.value <0.05) function screened the top5 marker genes of 9 types of cells (**Figure 2C**). We found that Fibroblast and Granulosa exhibited greater heterogeneity. Given that Granulosa is the primary cell type implicated in ovarian aging, we conducted a comprehensive investigation of Granulosa. Through the FindCluster function (resolution=0.2) we obtained 6 types of Granulosa (ARID5B+ Granulosa, GSTA1+ Granulosa, JUN+ Granulosa, MT-CO2+ Granulosa, HMGB1+ Granulosa, KRT18+ Granulosa; **Figures 2D, E**). The abundance of these Granulosa subtypes in ovarian tissue decreased sequentially. We performed functional enrichment analysis for the differentially expressed genes (p_val_adj<0.01, |log2FC|>1) of each subtype of Granulosa separately (differentially expressed genes Appendix; **Figure S1**). The results showed that the highly expressed genes in ARID5B+ Granulosa were mainly involved in the response to temperature stimulus, Electron transport chain: OXPHOS system in mitochondria, VEGFA-VEGFR2 signaling pathway and other signaling pathways. Its core regulatory network (MCODE) consists of EIF1, HSP90AA1, HSP90AB1, and HSPD1. The highly expressed genes in GSTA1+ Granulosa are mainly involved in signaling pathways such as response to unfolded protein, Attenuation phase, Cellular response to stress. Its core regulatory module consists of HSPA1A, HSPH1, HSP90AA1, HSPA8, DNAJB1, DNAJA1, EIF1, HSPD1, HSP90AB1, HSPE1. Highly expressed genes in JUN+ Granulosa are mainly involved in Host-pathogen interaction of human coronaviruses-MAPK signaling, regulation of hemopoiesis, negative regulation of transcription from RNA polymerase II promoter in response to stress. There are two core regulatory networks, which are HSPA1A, DNAJB1, HSPA1B; JUNB, JUN, FOS. The highly expressed genes in MT-CO2+ Granulosa are mostly engaged in the Electron transport chain: OXPHOS system in mitochondria, proton transmembrane transport, Cellular response to stress and other signaling pathways. Its primary regulatory module comprises of ND1, ND2, ND3, ND4, ND5, ND4L, COX1, COX2, COX3, CYTB. The highly expressed genes in HMGB1+ Granulosa are mainly involved in cell division, Cell cycle (Mitotic), Retinoblastoma gene in cancer and other signaling pathways. There are two core regulatory modules, which are respectively composed of CDK1, CDC20, CKS2, PTTG1, UBE2C, CCNB1, CKS1B; TUBB, TUBA1B, STMN1. The genes of these two modules can regulate cell division. The highly expressed genes in KRT18+ Granulosa are mainly involved in chaperone-mediated protein folding. These results reflect the functions of different types of Granulosa. Changes in the functional status of these follicular cells before and after perimenopause may have important implications for ovarian functional decline.

Differentiation trajectories of 6 types of follicular cells were constructed using CytoTRACE (**Figures 2F, G**). We found that the differentiation status of Granulosa was: ARID5B+ Granulosa-> JUN+ Granulosa-> KRT18+ Granulosa-> MT-CO2+ Granulosa-> GSTA1+ Granulosa-> HMGB1+ Granulosa (**Figure 2H**). The main genes involved in follicular cell differentiation are RBP1, TMSB10, SERPINE2, TMSB4X and IFI27. Their main functions are regulation of ATP-dependent activity, negative regulation of protein polymerization. The genes related to follicular cell stemness maintenance mainly include DCN, ARID5B, EIF1, HSP90AB1, STAR, etc., and their main functions are response to unfolded protein, chaperone-mediated protein complex assembly, etc. (**Figure 2I**). This result suggests that heat shock proteins produced by maintaining higher temperatures may promote the maintenance of follicular cell function in the ovary.

Estrogen changes around perimenopause are an important cause of perimenopausal syndrome (35). We obtained 7 Estrogen-related signaling pathways from the MSIGDB database. The activity of these 7 signaling pathways in cells was assessed by AddModuleScore. The results showed that GOBP_NEGATIVE_REGULATION_OF_INTRACELLULAR_ESTROGEN_RECEPTOR_SIGNALING_PATHWAY signaling pathway was significantly activated mainly in muscle cells, while HP_ABNORMAL_CIRCULATING _ESTROGEN_LEVEL was activated mainly in HMGB1+ Granulosa and GSTA1+ Granulosa (**Figure S2**). Considering that these two states of Granulosa are at the end stage of differentiation, we therefore hypothesize that the state of Granulosa at the end stage is associated with aberrant regulation of ESTROGEN levels.

### Changes in the microenvironment of ovarian tissue before and after perimenopause

**Figure 3:**
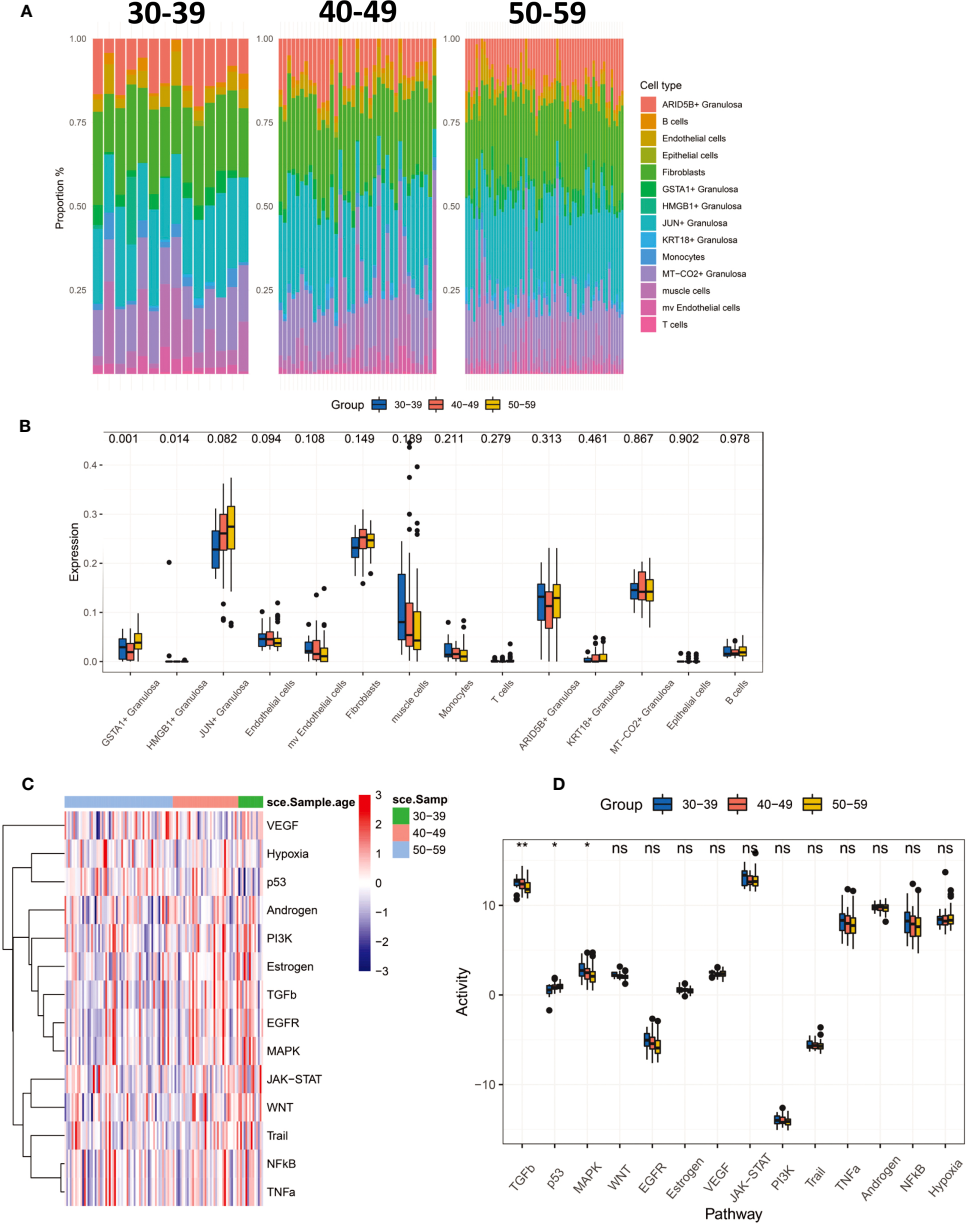
CIBERSORTx deconvolved 112 ovarian tissues, resulting in tissue infiltration scores for 14 cell types. **(A)** Percentage distribution of tissue infiltration scores for 14 cell types in three groups of 30-39, 40-49, 50-59. **(B)** Comparison of 14 cell types in three groups (Kruskal-Wallis test). **(C)** The Progeny algorithm assesses the activity of 14 signaling pathways in 112 samples. **(D)** The activity of 14 signaling pathways was compared among the three age groups (Kruskal-Wallis test). P<0.01**; P<0.05*.

To characterize the changing characteristics of the ovarian microenvironment in women before and after perimenopause, we deconvolved 112 ovarian tissues with CIBERSORTX and obtained scores for 14 cell types (**Figure 3A**). The results showed that GSTA1+ Granulosa and HMGB1+ Granulosa were significantly different between the three age groups (**Figure 3B**). Both types of Granulosa were noted to be in the terminal differentiation phase and were significantly higher in the 50-59 age group. At the same time, we noted a progressive increase in JUN+ Granulosa scores with age, as well as a progressive increase in mv Endothelial cells and Muscle cells. Although not significant, it still reflects the dynamic trend of these cells before and after menopause. We inferred the signaling pathway activity of 112 ovarian samples by Progeny (**Figures 3C, D**). The results showed that the activity of TGFb and MAPK signaling pathways significantly decreased with age, while the activity of p53 signaling pathway significantly increased. This result suggests that the decrease in TGFb and MAPK signaling pathway activity and the increase in p53 signaling pathway activity are the main features of the changes around perimenopause.

### Weighted gene co-expression network analysis of the tissue of the ovary before and after perimenopause

**Figure 4:**
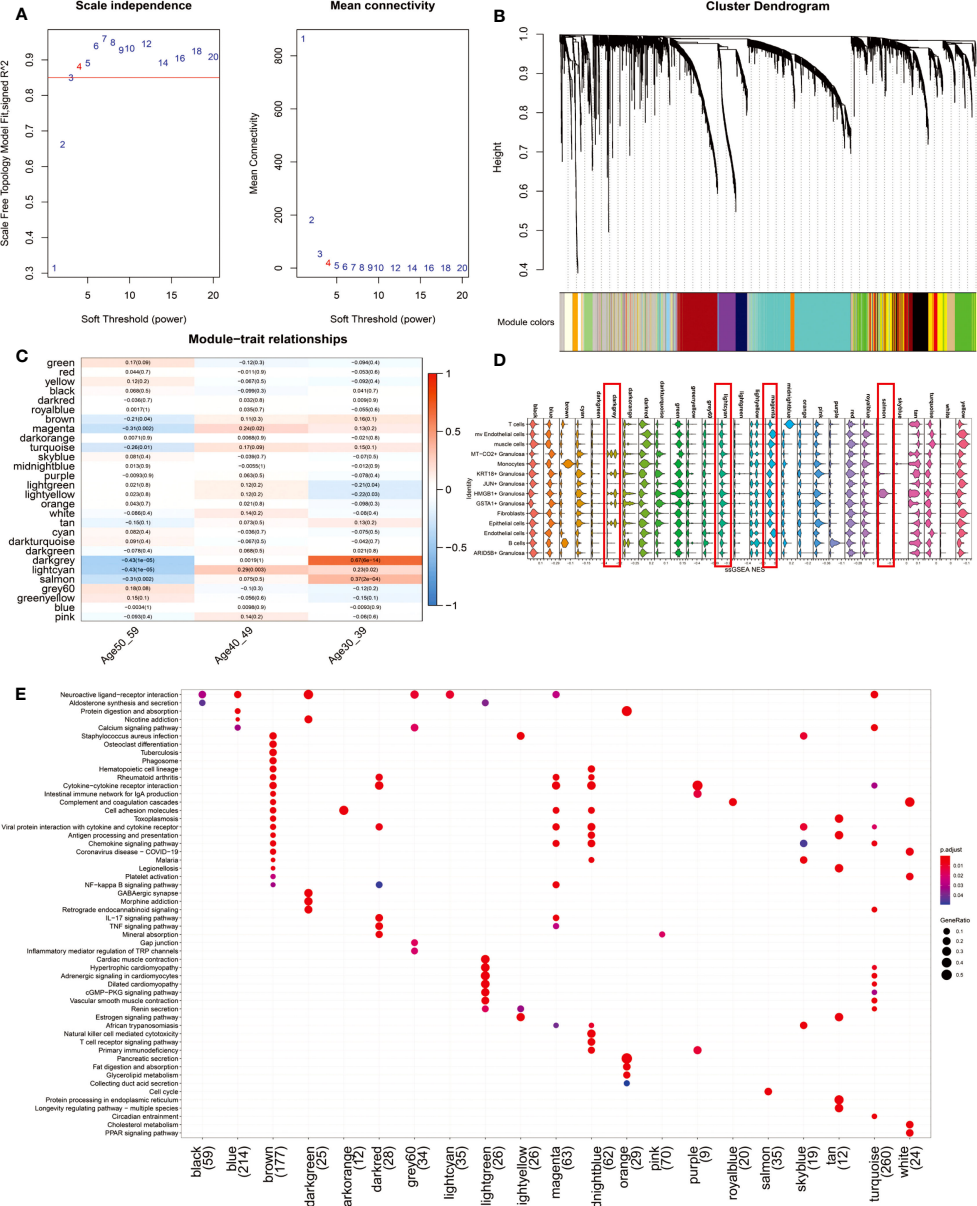
WGCNA results of 112 ovarian tissue samples. **(A)** To fit the network to a scale-free distribution, a soft threshold of 4 was chosen. **(B)** Twenty-nine co-expressed regulatory modules were obtained with an overhang fusion distance of 0.25. **(C)** Heatmap of Module-trait relationships. From the figure, it can be found that magenta, lightcyan modules are related to the 40-49 age group, while darkgrey, lightcyan and salmon modules are related to the 30-39 age group. **(D)** The gene set consisting of 29 modules of hub genes (R>0.3, p<0.001) was mapped to single-cell sequencing data by the ssGSEA algorithm. We focused on comparing the enrichment scores of darkgrey, lightcyan, magenta, and salmon on four modules in 14 cell types. **(E)** KEGG enrichment results of the 21-module hub genes. Eight of the 29 modules were not enriched for a statistically significant signaling pathway (p.adjust<0.05).

To further explore the gene co-expression profile of the ovary before and after perimenopause, we used WGCNA. 29 co-expression networks (**Figure 4B**) were eventually obtained by selecting a soft threshold of 4 (**Figure 4A**). The magenta, lightcyan modules were positively correlated with the 40-49 age group (magenta: R=0.24, p=0.02; lightcyan: R=0.29, p=0.003), whereas the darkgrey and salmon modules were significantly positively correlated with the 30-39 age group (darkgrey: R= 0.67, p<0.001; salmon: R=0.37, p<0.001) (**Figure 4C**). We constituted these 29 modules of hub genes (R>0.3, p<0.001) into a 29-gene set that was mapped onto a single-cell transcriptional profile using the ssGSEA algorithm (**Figure 4D**). The results showed that darkgrey and lightcyan modules were mainly enriched in MT-CO2+ Granulosa, KRT18+ Granulosa, HMGB1+ Granulosa, GSTA1+ Granulosa and Epithelial cells. The hub genes of the Lightcyan module were mainly enriched in Neuroactive ligand-receptor interaction. Magenta module was mainly enriched in mv Endothelial cells and Endothelial cells, and their main functions are Cytokine-cytokine receptor interaction, Cell adhesion molecules, NF-kappa B signaling pathway, IL-17 signaling pathway and other signaling pathways (**Figure 4E**). The results suggest that vascular inflammation is a major feature of perimenopausal syndrome in the age group 40-49. Salmon modules were predominantly enriched in HMGB1+ Granulosa cells, whose primary function is the Cell cycle. This result suggests that activation of cell cycle signaling pathways is a major feature of HMGB1+ Granulosa in the age group 30-39.

### Patterns of gene expression and transcription factor activity changes in the ovaries of women in the pre-perimenopausal period (40-49 vs. 30-39)

**Figure 5:**
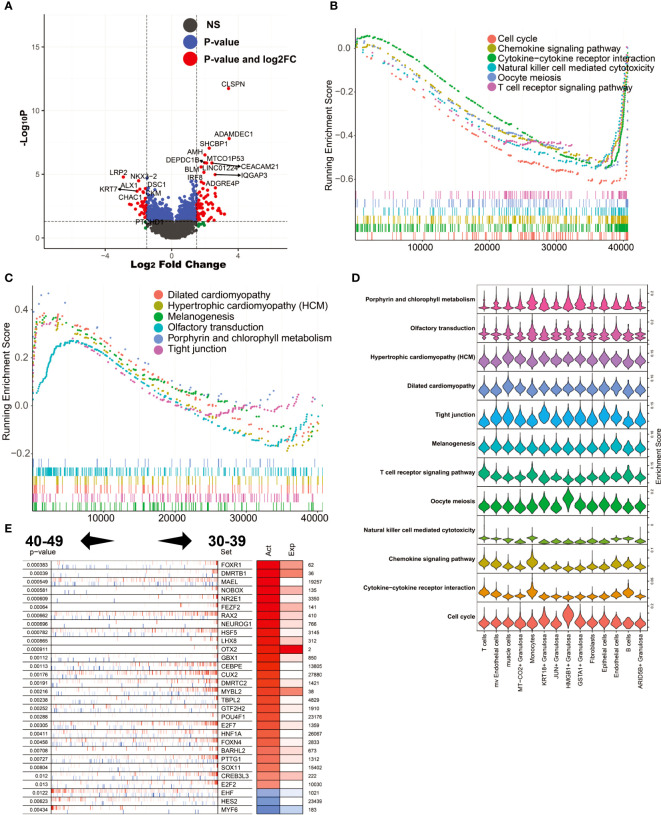
Analysis of differentially expressed genes and master regulators of 40-49 vs. 30-39. **(A)** Volcano plot of differentially expressed genes. **(B)** GSEA analysis (log2FC ranking), signaling pathways enriched in the 30-39 age group. **(C)** GSEA analysis, signaling pathways enriched in the 40-49 age group. **(D)** Mapping of signaling pathways enriched in the two subgroups to single-cell sequencing data. **(E)** Results of master regulator analysis.

During perimenopause, many changes occur in the cellular and physiological functions of the ovary. To explore the molecular mechanisms underlying these changes, we performed differential expression analysis of ovarian tissues from the 40-49 age group and the 30-39 age group. The results showed that CLSPN, ADAMDEC1, SHCBP1, AMH and MTCO1P53 were highly expressed in the 40-49 age group, while LRP2, KRT7, ALX1, CHAC1 and DSC1 were highly expressed in the 30-39 age group (**Figure 5A**). GSEA was used to enrich the MSIGDB KEGG signaling pathway in both groups (ranked according to log2FC; **Figures 5B, C**). The results showed that the enriched signaling pathways in the ovaries of the 30-39 age group included Cell cycle, Chemokine signaling pathway, Cytokine-cytokine receptor interaction, Natural killer cell mediated cytotoxicity, Oocyte meiosis, and T cell receptor signaling pathway. The enriched signaling pathways in the ovary of the 40-49 age group include Dilated cardiomyopathy, Hypertrophic cardiomyopathy(HCM), Melanogenesis, Olfactory transduction, Porphyrin and chlorophyII metabolism, Tight junction. We mapped these signaling pathways onto a single-cell sequencing map. The results showed that Cell cycle was mainly enriched in HMGB1+ Granulosa; Cytokine-cytokine receptor interaction was mainly enriched in T cells, Monocytes and B cells; Chemokine signaling pathway was mainly enriched in T cells and Monocytes; Oocyte meiosis was mainly enriched in HMGB1+ Granulosa Tight junction was mainly enriched in KRT18+ Granulosa; Dilated cardiomyopathy was mainly enriched in muscle cells (**Figure 5D**). These results reflect the precise cytological localization of these enriched signaling pathways. Further, we explored the master regulators driving perimenopause. The results showed that the main regulators in the 30-39 age group were FOXR1, DMRTB1, MAEL, NOBOX, NR2E1, etc., and the main regulators in the 40-49 age group were MYF6, HES2, EHF (**Figure 5E**). FOXR1 is critical for determining cell fate during early embryonic development (36) and is essential for female fertility.

### Changes in gene expression and transcription factor activity in female ovaries after perimenopause (50-59 vs. 40-49)

**Figure 6:**
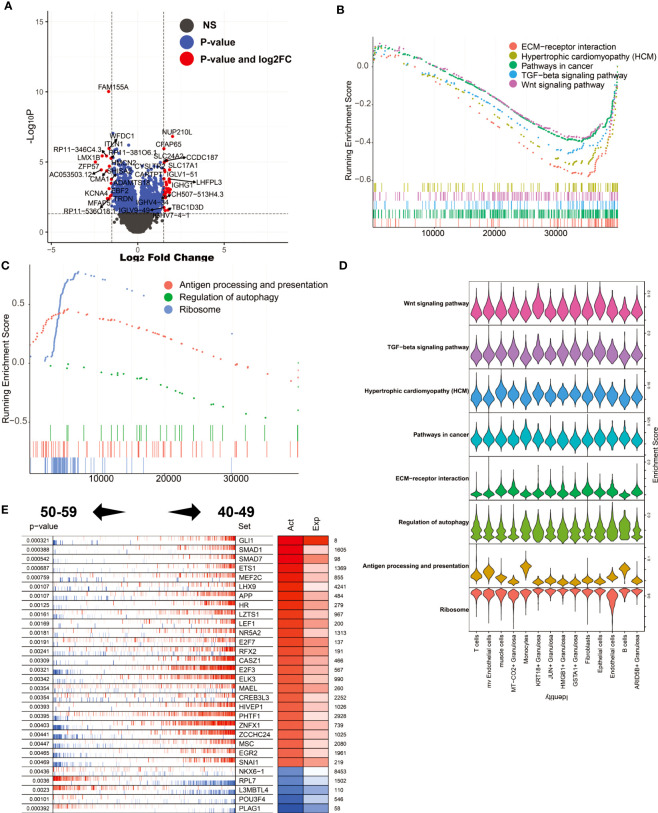
Analysis of differentially expressed genes and master regulators of 50-59 vs. 40-49. **(A)** Volcano plot of differentially expressed genes. **(B)** GSEA analysis (log2FC ranking), signaling pathways enriched in the 40-49 age group. **(C)** GSEA analysis, signaling pathways enriched in the 50-59 age group. **(D)** Mapping of signaling pathways enriched in the two subgroups to single-cell sequencing data. **(E)** Results of master regulator analysis.

After perimenopause, ovarian function decline is a physiological phenomenon unique to human women. To explore the molecular biological mechanism of ovarian decline, we compared the differentially expressed genes in ovarian tissue between the 50-59 age group and the 40-49 age group (**Figure 6A**). The results showed that NUP210L, CFAP65, SLC24A2, CCDC187 and other genes were significantly increased in the 50-59 age group, while FAM155A, WFDC1, ITLN1, ZFP57, KCNA4 and other genes were significantly decreased. We noticed that immunoglobulin-related genes such as IGLV-51, IGHG1, IGHV4-34, IGLV9-48, IGHV7-4-1 were significantly elevated in the 50-59 age group. This result suggests that plasma cells play an important role in the progressive decline of the ovary after perimenopause. Through GSEA, we found that the enriched signaling pathways in the 40-49 age group mainly include ECM-receptor interaction, Hypertrophic cardiomyopathy (HCM), Pathways in cancer, TGF-beta signaling pathway, Wnt signaling pathway. In the 50-59 age group, Antigen processing and presentation, Regulation of autophagy, Ribosome were significantly enriched (**Figures 6B, C**). This result suggests that immune responses and autophagy may be major factors in ovarian decline. Again, we mapped these signaling pathways to single-cell sequencing data. The results showed that Antigen processing and presentation were mainly enriched in mv Endothelial cells, Monocytes, and B cells (**Figure 6D**). It is worth noting that activation of inflammation-related signaling pathways by mv Endotheilal cells is a major cause of microangiopathies. Next, we also analyzed the major master regulators driving ovarian decline. We found that the master regulators in the 40-49 age group are GLI1, SMAD1, SMAD7, ETS1, MEF2C, etc., while the master regulators in the 50-59 age group are PLAG1, POU3F4, L3MBTL4, RPL7, NKX6-1 (**Figure 6E**).

### Transcription factor activity is used to diagnose the status of ovary in women before and after menopause

**Figure 7:**
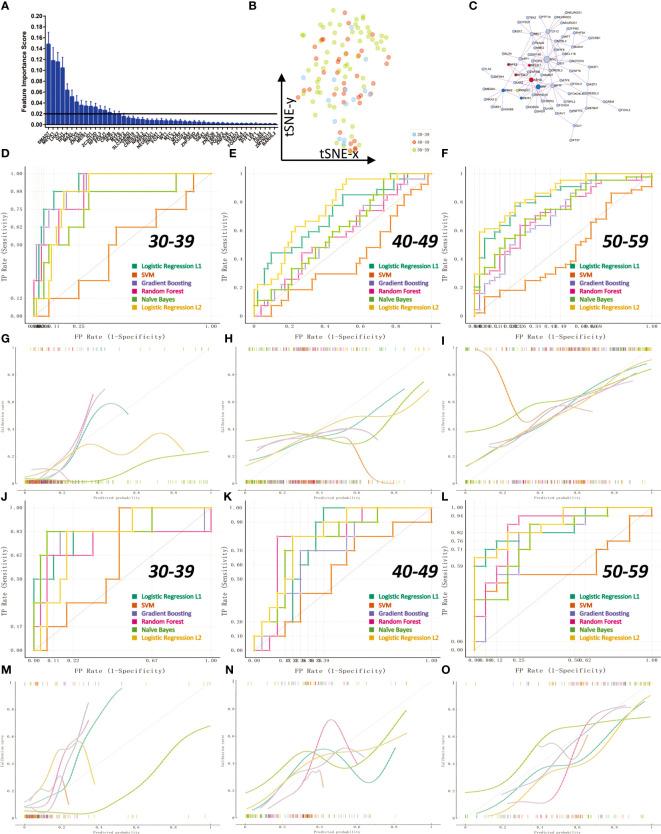
Transcription factor activity for diagnosis of ovarian status in pre- and post-menopausal women. The 112 samples were divided into training set and validation set according to the 70% cutoff point. **(A)** Through 1000 times of Logistic Regression L1, 16 transcription factors with an average feature weight higher than 0.2 were screened. **(B)** Non-linear dimensionality reduction (t-SNE algorithm) of 112 ovarian samples using 16 transcription factors. **(C)** PPI network regulation map of 105 transcription factors with average weights other than 0. There are two core regulation modules (MCODE algorithm), the red module is composed of NFE2, NFE2L1, NFE2L3, ASH2L; the blue module is composed of PBX2, SRF, MEIS1. **(D, G)** ROC curve and calibration curve of training set 10-fold cross-validation at age 30-39. **(E, H)** ROC curve and calibration curve of 10-fold cross-validation in the training set at age 40-49. **(F, I)** ROC curve and calibration curve of training set 10-fold cross-validation at age 50-59. **(J, M)** ROC curve and calibration curve of the validation set at age 30-39. **(K, N)** ROC curves and calibration curves of the validation set at age 40-49. **(L, O)** ROC curves and calibration curves of the validation set at age 50-59.

Compared with the information of gene expression level, ovarian transcription factor activity can better reflect the real state of ovarian tissue. We constructed a classification model for diagnosing ovarian status before and after perimenopause through machine learning algorithms. First, we calculated the weight information of transcription factors through 1000 logistic regression (L1) times, and screened 16 transcription factors with weights greater than 0.2 from them for subsequent model construction (**Figure 7A**; The weight information is stored in Appendix: Feature Importance LR L1). These 16 transcription factors could not get clear boundaries on the t-SNE 2D scatter plot (**Figure 7B**). We constructed a PPI network for 105 transcription factors with non-zero weights (**Figure 7C**). We constructed six different machine learning models (SVM lib, Random Forest, Naive Bayes, Logistic Regression L2, Logistic Regression L1, Gradient Boosting). We divided the samples into a training set and a test set according to 70%. Ten-fold cross-validation (Stratified) was used to train the model in the training set. The results showed that Logistic Regression L2 had the best average model performance (AUC=0.82, F1 = 0.66). We compared the performance of the models in predicting the three groups separately (**Figures 7D–F**). The results showed that all models performed poorly in the prediction of the 40-49 age group (AUC<0.8). The results of the calibration curves also reflect the previous conclusions (**Figures 7G–I**). Next, we compared the models in the validation set. The results show that Logistic Regression L2 also has the best performance in the test set (AUC=0.84, F1 = 0.57). Logistic Regression L2 was again the best model in terms of predictive performance for all three subgroups (**Figures 7J–L**). The calibration curves for the validation set reflect the same results (**Figures 7M–O**). It illustrates that our Logistic Regression L2 model constructed from 16 transcription factor activity profiles is able to diagnose the state of the ovary in pre- and post-perimenopausal women. The data obtained by the machine learning model analysis is in Appendix: Machine Learning Models Results.

## Discussion

The normal menstrual cycle is regulated by the hypothalamic pituitary ovarian gonadal axis (37). After menstruation, the ovaries secrete estrogen, which encourages the development of follicles to form the follicular phase, during which estrogen does not cause the body temperature to rise (38). After menopause, ovarian function declines. A group of syndromes caused by fluctuations or decreases in sex hormones around the time of menopause, mainly dysfunction of the autonomic nervous system, accompanied by neuropsychological symptoms, is called perimenopausal syndrome or menopause syndrome (39). In this study, we explored the molecular biology and cell biology of ovarian tissue before and after perimenopause using transcriptomic data from the ovaries of women aged 30-59 combined with single-cell sequencing data from the ovaries. We identified six subtypes of follicular cells in the ovary and constructed their differentiation trajectories (ARID5B+ Granulosa -> JUN+ Granulosa -> KRT18+ Granulosa -> MT-CO2+ Granulosa -> GSTA1+ Granulosa -> HMGB1+ Granulosa). The genes that drive Granulosa differentiation (RBP1, TMSB10, SERPINE2, TMSB4X, etc.) are mainly enriched in the regulation of ATP-dependent activity signaling pathway, while the genes that maintain the Granulosa state (DCN, ARID5B, EIF1, HSP90AB1 etc.) are mainly enriched in the response to unfolded protein and chaperone-mediated protein complex assembly signaling pathways. In addition, we found significantly higher GSTA1+ Granulosa and HMGB1+ Granulosa content in follicular cells in the 50-59 age group. These results suggest that the Granulosa content at the terminal end of differentiation is significantly increased after perimenopause. We found that signaling pathways involved in heat shock proteins play an important role in early differentiation and identified highly expressed genes in ARID5B+ Granulosa that are mainly involved in the response to temperature stimulus signaling pathway. Considering that ovarian temperature rises significantly during ovulation, the activity of heat shock protein-related signaling pathways is significantly increased under these conditions. Therefore, we suggest that the failure to increase ovarian temperature cyclically after menopause may be responsible for ovarian decline. The use of mugwort in Chinese medicine to delay menopause and thus indirectly protect a woman’s state of health may be related to this mechanism (40). By progeny analysis, we found that the activity of the TGFb and MAPK signaling pathways decreased progressively with menopause, while the activity of the p53 signaling pathway increased progressively. The TGFb signaling pathway has been shown to play a key role in the ovarian primordial follicle pool. Blockade of the TGFb signaling pathway is directly associated with a variety of female reproductive diseases (41). P53 is a tumor suppressor gene, which can prevent the occurrence of cancer during ovarian recession (42).

Further, we explored the gene expression profile of the ovaries around perimenopause (30–39, 40–49, 50–59) using WGCNA. We found that the megena module associated with the 40-49 age group was significantly enriched in mv Endothelial cells and was mainly involved in the inflammation-related signaling pathway (Cytokine-cytokine receptor interaction, NF-kappa B signaling pathway). It indicates the beginning of inflammatory apoptosis of the blood vessels of the ovaries at this age. The perimenopausal syndrome in women mainly occurs at this stage. In addition, we found that the salmon module associated with the 30-39 age group was significantly enriched in HMGB1+ Granulosa, mainly involved in the Cell Cycle signaling pathway. It suggests a rapid proliferation rate of HMGB1+ Granulosa in the 30-39 age group. This was not the case in other age groups. To gain insight into the process of gene expression regulation changes in the ovary before and after perimenopause, we first compared gene expression differences and differences in master regulators between the 40-49 age group and the 30-39 age group. We found that inflammation-related signaling pathways in the 30-39 age group were mainly activated in T cells, Monocytes and B cells, while the Cell cycle was mainly activated in HMGB1+ Granulosa. This result is consistent with our previous analysis (43). Dilated Cardiomyopathy, Hypertrophic Cardiomyopathy (HCM) in muscle cells was significantly higher in the 40-49 age group. This is associated with the hypertensive symptoms typical of perimenopausal syndrome (44). By master regulator analysis, we found that transcription factors such as FOXR1, OTX2, MYBL2, HNF1A, FOXN4 were significantly activated in the 30-39 age group, while MYF6, HES2, EHF were significantly activated in the 40-49 age group. FOXR1, FOXN4, OTX2 have been shown to play a key role in germ cell development (45). The loss of activity of these transcription factors represents a loss of reproductive function of the follicle. Next, we compared differences in gene expression and differences in master regulators between the 50-59 age group and the 40-49 age group. We found significantly higher expression of immunoglobulin-related genes in the 50-59 age group than in the 40-49 age group. The result suggests that plasma cells in the 50-59 age group may play an important role in the natural decline of the ovary. We found that the signaling pathways GLI1, SMAD1, SMAD7, APP, and EGR1 were significantly activated in the 40-49 age group, whereas PLAG1, POU3F4, L3MBTL4, RPL7, and NKX6-1 were significantly activated in the 50-59 age group. Activation of the TGFb signaling pathway can upregulate its own signaling via SMAD7, which is essential for normal folliculogenesis (46).

Finally, we constructed diagnostic models for determining ovarian status before and after perimenopause. We found 16 markers of transcription factor activity that could be used to diagnose ovarian status. We found that our Logistic Regression L2 model obtained a mean AUC=0.82, F1 = 0.66 in the training set (10-fold cross-validation). In the validation set, we obtained a mean AUC=0.84, F1 = 0.51. This model can be used to determine the ovarian status of women around perimenopause.

This study has limitations. We attempted to define pre- and post-perimenopause in terms of age by dividing the sample into three groups. This stratification assumes a relationship between hormonal status and age, however there is still no well-established method for measuring hormonal status in humans (47). Therefore, perimenopause is difficult to define. Using age as a proxy for hormonal status is more challenging (48). Despite the ambiguity of this analysis, the use of age to define perimenopause remains a widely accepted but relatively less accurate method, and the diagnostic models developed by this method may provide inspiration for future studies. In addition, we assessed the activity of Estrogen-related signaling pathways at the single-cell level and found significant activation of Estrogen level-disordered signaling pathways in terminally differentiated Granulosa. Considering the higher proportion of terminally differentiated Granulosa in the 50-59 age group, these results suggest, to some extent, that disturbances in Estrogen around perimenopause are associated with aging in Granulosa. However, more experimental evidence is still needed to verify these findings.

In conclusion, we have used single-cell sequencing data from ovarian tissue and transcriptome sequencing data to deeply explore the cellular biology and molecular biology of the female ovary before and after perimenopause. These results will help us to better understand the molecular mechanisms underlying perimenopausal syndrome in human women and provide a theoretical basis for the development of perimenopausal care protocols. The results of these studies still need to be corroborated by more clinical data.

## Data availability statement

The original contributions presented in the study are included in the article/**Supplementary Material**. Further inquiries can be directed to the corresponding authors.

## Author contributions

QL: Methodology, Software, Validation, Formal analysis, Investigation, Writing-Original Draft, Data Curation, Supervision. ZY: Writing- Review and Editing, Visualization, Supervision, Methodology. FW: Writing- Review and Editing, Formal analysis, Investigation, Methodology. JW: Software, Validation. HL: Writing- Review and Editing, Formal analysis. HZ: Conceptualization, Project administration. ML: Visualization, Methodology. KL: Software, Validation. All authors contributed to the article and approved the submitted version.

## Supplementary material

Supplementary Figure 1 Results of functional enrichment analysis of differentially expressed genes in six Granulosa cell subtypes. (A) Results of functional enrichment analysis of highly expressed genes in ARID5B+ Granulosa. (B) Results of functional enrichment analysis of highly expressed genes in GSTA1+ Granulosa. (C) Results of functional enrichment analysis of highly expressed genes in JUN+ Granulosa. (D) Results of functional enrichment analysis of highly expressed genes in MT-CO2+ Granulosa. (E) Results of functional enrichment analysis of highly expressed genes in HMGB1+ Granulosa. (F) Results of functional enrichment analysis of highly expressed genes in KRT18+ Granulosa.

Supplementary Figure 2 Activity of Estrogen-related signaling pathways at the single-cell level. The Estrogen-Related Signaling Pathways was obtained from the MSIGDB database.
