# Supplementary figures and images for: Molecular mechanisms regulating natural menopause in the female ovary: a study based on transcriptomic data
Source: Front Endocrinol (Lausanne). 2023 Jul 24;14:1004245. doi: 10.3389/fendo.2023.1004245 (PMC10411606; doi:10.3389/fendo.2023.1004245)

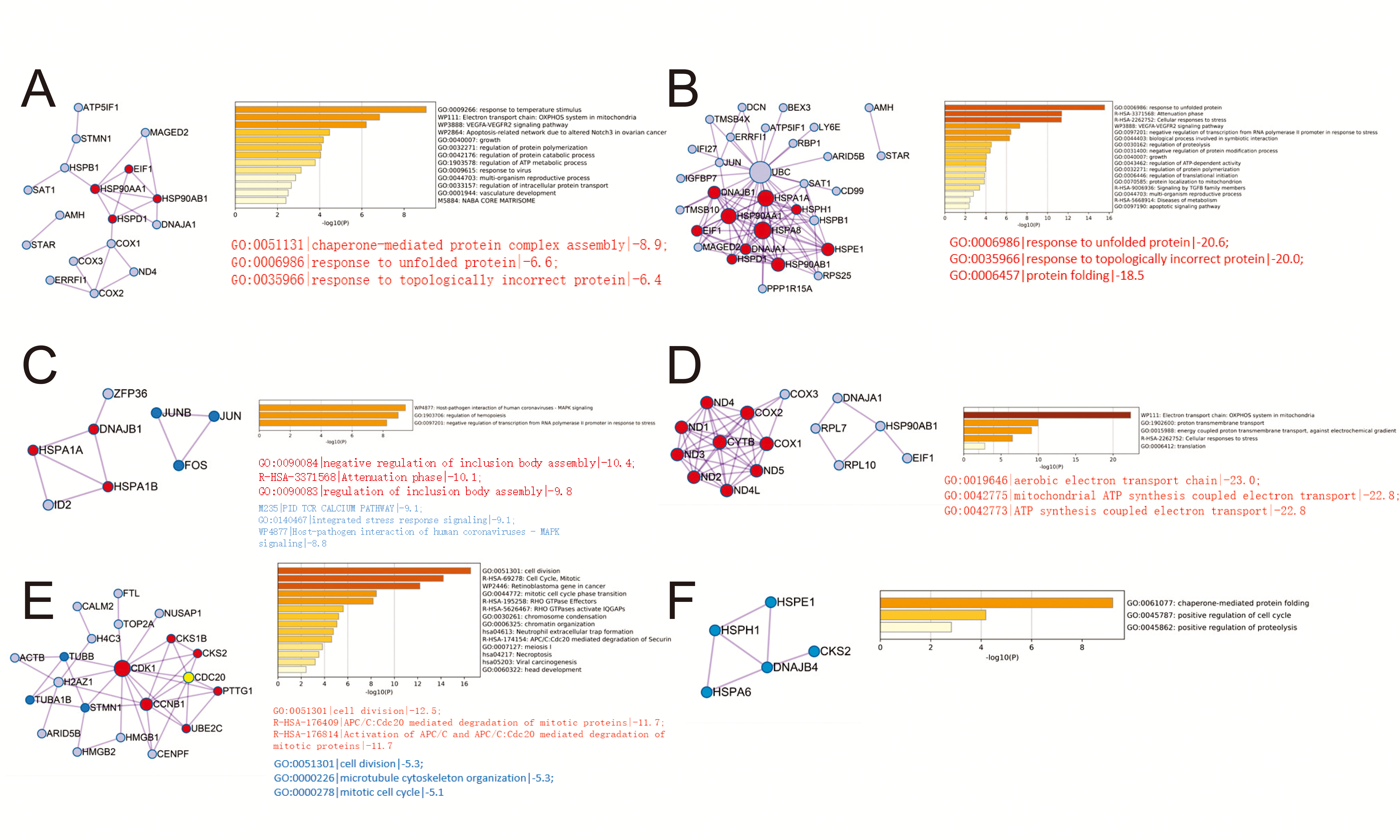

Supplement: Supplementary Figure 1 — Results of functional enrichment analysis of differentially expressed genes in six Granulosa cell subtypes. (A) Results of functional enrichment analysis of highly expressed genes in ARID5B+ Granulosa. (B) Results of functional enrichment analysis of highly expressed genes in GSTA1+ Granulosa. (C) Results of functional enrichment analysis of highly expressed genes in JUN+ Granulosa. (D) Results of functional enrichment analysis of highly expressed genes in MT-CO2+ Granulosa. (E) Results of functional enrichment analysis of highly expressed genes in HMGB1+ Granulosa. (F) Results of functional enrichment analysis of highly expressed genes in KRT18+ Granulosa. [file Image_1.jpeg]

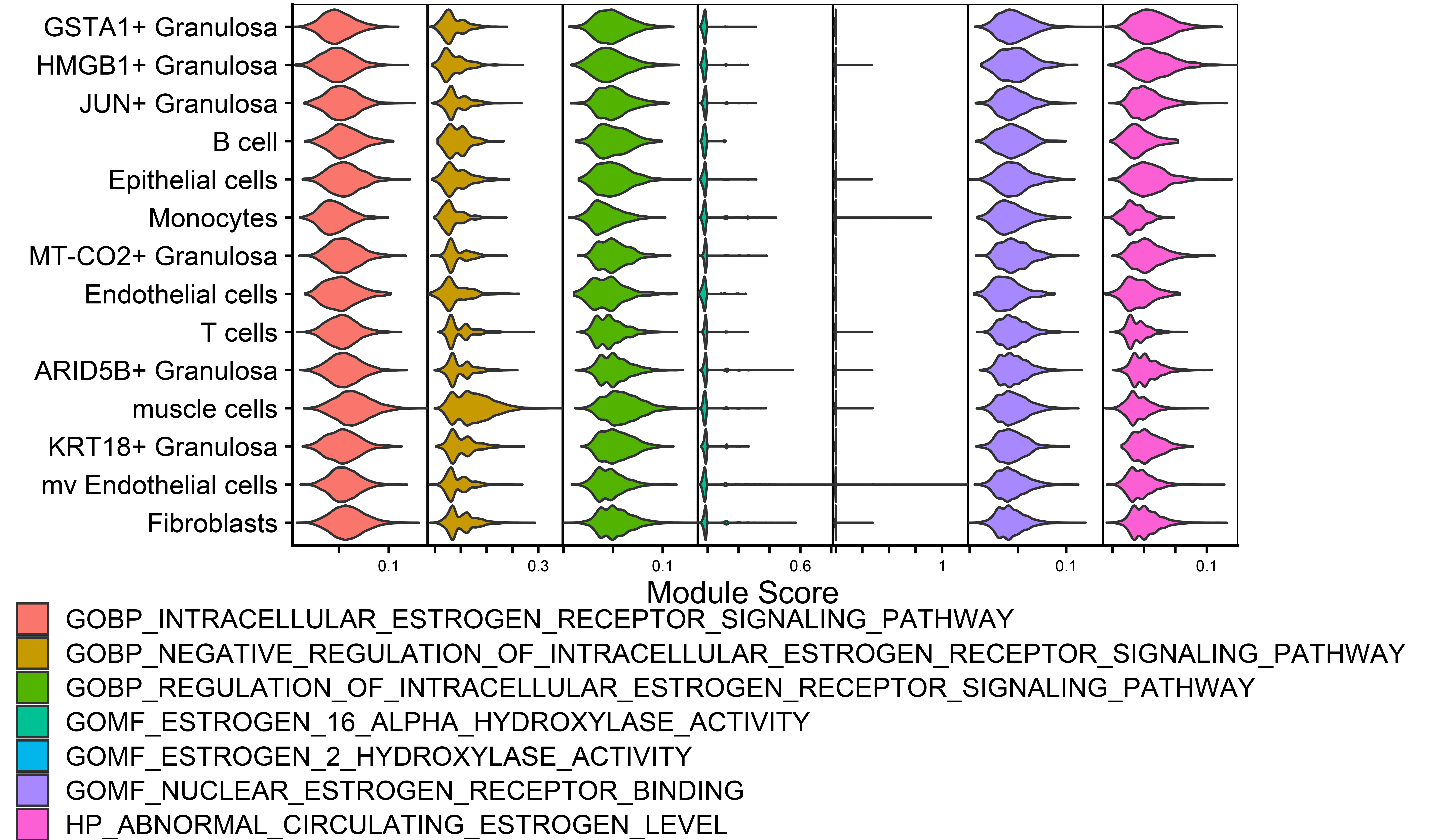

Supplement: Supplementary Figure 2 — Activity of Estrogen-related signaling pathways at the single-cell level. The Estrogen-Related Signaling Pathways was obtained from the MSIGDB database. [file Image_2.jpeg]
